# Acceptability and Feasibility of Wearable Transdermal Alcohol Sensors: Systematic Review

**DOI:** 10.2196/40210

**Published:** 2022-12-23

**Authors:** Eileen Brobbin, Paolo Deluca, Sofia Hemrage, Colin Drummond

**Affiliations:** 1 Department of Addictions Institute of Psychiatry, Psychology & Neuroscience King's College London London United Kingdom

**Keywords:** alcohol consumption, alcohol monitoring, digital technology, transdermal alcohol sensors, wearables, acceptability, feasibility, monitoring, sensors, real-time feedback, health promotion, alcohol intake

## Abstract

**Background:**

Transdermal alcohol sensors (TASs) have the potential to be used to monitor alcohol consumption objectively and continuously. These devices can provide real-time feedback to the user, researcher, or health professional and measure alcohol consumption and peaks of use, thereby addressing some of the limitations of the current methods, including breathalyzers and self-reports.

**Objective:**

This systematic review aims to evaluate the acceptability and feasibility of the currently available TAS devices.

**Methods:**

A systematic search was conducted in CINAHL, EMBASE, Google Scholar, MEDLINE, PsycINFO, PubMed, and Scopus bibliographic databases in February 2021. Two members of our study team independently screened studies for inclusion, extracted data, and assessed the risk of bias. The study’s methodological quality was appraised using the Mixed Methods Appraisal Tool. The primary outcome was TAS acceptability. The secondary outcome was feasibility. The data are presented as a narrative synthesis.

**Results:**

We identified and analyzed 22 studies. Study designs included laboratory- and ambulatory-based studies, mixed designs, randomized controlled trials, and focus groups, and the length the device was worn ranged from days to weeks. Although views on TASs were generally positive with high compliance, some factors were indicated as potential barriers and there are suggestions to overcome these.

**Conclusions:**

There is a lack of research investigating the acceptability and feasibility of TAS devices as a tool to monitor alcohol consumption in clinical and nonclinical populations. Although preliminary evidence suggests their potential in short-term laboratory-based studies with volunteers, more research is needed to establish long-term daily use with other populations, specifically, in the clinical and the criminal justice system.

**Trial Registration:**

PROSPERO CRD42021231027; https://www.crd.york.ac.uk/prospero/display_record.php?RecordID=231027

## Introduction

### Background

In recent years, there have been advances in the development of various wearable transdermal alcohol sensor (TAS) devices. These devices measure alcohol consumption from vapors off the skin via sweat, known as transdermal alcohol concentration. The advantage of these devices is the possibility to wear them all day, allowing for long-term continuous data capture [1] and recording drinking episodes accurately in near real time. These are features that overcome some of the limitations of other methods. Breath and urine analysis is limited by the short half-life of ethanol [2]. Blood markers of heavy alcohol consumption (eg, gamma-glutamyl transferase, liver function tests, mean cell volume, carbohydrate-deficient transferrin) have limitations in terms of sensitivity or specificity [2,3]. Although other alcohol biomarkers such as phosphatidyl ethanol found in blood and ethyl glucuronide and ethyl sulfate found in urine have been found to have high sensitivity and specificity, these tests can only be used to detect if alcohol has been consumed within the last number of days [4-6]. Further, all these tests require administration by a trained health professional. In contrast, TAS devices are noninvasive, objective, easy-to-use, low-maintenance, and allow for the study of behaviors in real-world contexts for potentially weeks or months at a time [7].

The newer TAS devices have the potential to communicate wirelessly through a sim card or with a smartphone [7]. Using this as a method of data collection could reduce the time and resources required for data capture. There is also the possibility for information to be uploaded over a mobile network and delivered to the patient, health professional, or researcher in near real time. Evidence suggests there is a slight time lag between drinking and peak transdermal alcohol concentration [7-10], but preliminary studies with newer generation devices have demonstrated a reduction in this time lag [7,9]. SCRAM is a chunkier device with various types worn on the ankle and is similar in appearance to a house arrest monitor, whereas WrisTAS, BACtrack, ION, and Quantac Tally are worn exclusively on the wrist and are smaller in style, closer in appearance to a FitBit, health watch, or pedometer, and are approximately the same size as a watch (information along with an image of each device is summarized in Multimedia Appendix 1).

### Potential Uses of TAS Devices

#### Alcohol Treatment and Interventions

TAS devices have the potential to improve clients’ engagement, increase clinicians’ ability to accurately assess consumption, and trigger real-time interventions. If relapses are caught early, the treatment service could personalize treatment and interventions for the client to prevent further or larger relapses [11]. In addition, TASs can capture regular data, which can be linked with contingency management (CM) for an effective treatment option [12-14].

#### Forensic Monitoring

The South Dakota 24/7 Sobriety initiative enforced alcohol monitoring for driving under the influence offenders. The use of TASs, breathalyzers, and sanctions for breaches was found to be effective. This project included 17,000 individuals between 2005 and 2010, and since then, has been extended to domestic violence and drug offences [15]. There has been some preliminary research implementing remote alcohol monitoring within the United Kingdom [16]. Most recently, England and Wales announced new legislation, where alcohol-related offenders may be banned from drinking alcohol and be ordered to wear a TAS for up to 120 days.

#### Research

Alcohol research mostly relies on retrospective self-report data. There is some evidence of the reliability of self-report [17]; however, it can be subject to bias [18,19]. Evidence suggests that alcohol consumption tends to be underreported [20] and may be more greatly underreported with nonroutine drinking patterns and heavy drinkers [21], whereas TASs could provide an objective measure.

#### Public Use

Newer TAS devices are designed for consumer use. They could be used to monitor alcohol levels before driving, as proof of sobriety at bars or public events, and be a diary for those interested in monitoring alcohol consumption for general health.

### Acceptability and Feasibility of TAS Devices

The acceptability and feasibility of health care interventions are important issues to consider in their development, evaluation, and implementation [22]. Although previous studies [7,12,23-25] have alluded to the acceptability and feasibility of TASs to objectively monitor alcohol consumption, there are a limited number of studies addressing this, and to our knowledge, there are no systematic reviews specifically investigating this. In this review, we consider acceptability as the device being perceived as appropriate, which is based on both cognitive and emotional responses to the devices and that this acceptability can be assessed before, during, or after wearing the device [22]. We consider feasibility as the extent to which this device could be implemented practically within the identified setting. This systematic review investigates the current knowledge by systematically identifying and evaluating the existing literature on the use of TAS devices in clinical and nonclinical populations, alone or in conjunction with a psychosocial intervention. The objective of this review was to assess the acceptability and feasibility of TAS devices with an overarching objective of establishing the barriers and facilitators to implementing these devices.

## Methods

### Systematic Review Registration

This systematic review has been written according to the PRISMA-P (Preferred Reporting Items for Systematic review and Meta-Analysis for Protocols) guidelines [26]. This protocol has been registered on the Prospective Register of Systematic Reviews (PROSPERO CRD42021231027). On review of the results, it was decided that the findings of this systematic review should be reported in 2 papers: this paper focusing on acceptability and feasibility outcomes and another paper on accuracy outcomes [27].

### Inclusion Criteria for the Studies

Studies meeting all the following criteria were included: full text, original studies, published in peer-reviewed journals, written in English, using a wearable transdermal sensor device(s) or investigating attitudes and experience of TAS use reporting acceptability or feasibility outcomes. There were no restrictions on publication year or participant clinical characteristics. Data based on conference abstracts, dissertations, and grey literature were not included.

### Information Sources

Bibliographic databases included CINAHL, EMBASE, Google Scholar, MEDLINE, PsycINFO, PubMed, and Scopus. Searches were carried out February 2021 (Textbox 1 and Multimedia Appendix 2 and Multimedia Appendix 3). The searches were supplemented by cross-checking the reference lists of key publications, related systematic reviews, and all included papers.

All identified titles and abstracts were screened in Covidence. From this list, the full text was retrieved and assessed for eligibility (EB). Any queries were discussed with a second reviewer (SH). Any disagreement was resolved through discussion with a third reviewer (PD). A data extraction form was created, piloted, and refined as necessary. EB extracted the data independently (Multimedia Appendix 4) and the second reviewer completed entries check for accuracy.

**Search terms.**

- Transdermal alcohol sensor
- Transdermal alcohol sensor device
- Transdermal alcohol monitoring device
- Transdermal alcohol bracelet
- Transdermal alcohol wristband
- Transdermal alcohol ankle
- Transdermal alcohol concentration
- Transdermal alcohol concentration data
- Transdermal alcohol sensor data
- Transdermal alcohol validity
- Transdermal alcohol acceptability
- Transdermal alcohol feasibility

### Outcomes in the Studies

All outcome measures reported in the included studies were extracted, including both objective and self-reported measures. The definition of acceptability outcomes for TASs in the context of this study are factors that affect participant willingness to use the device in treatment or rehabilitation. The definition of feasibility outcomes for TASs in the context of this study are factors that would impact the introduction, including the operational capability of these devices in alcohol treatment services as part of treatment and individual skills required by wearers.

### Quality Assessment

We used the Mixed Methods Appraisal Tool (MMAT), as it was designed for the appraisal in reviews that include a range of designs (qualitative, quantitative, and mixed methods) [28]. For each included study, we determined the study design and then applied the appropriate screening criteria; this provided an overall quality score for the study [28]. EB independently completed the appraisal, and queries were discussed with a second reviewer (SH). Any disagreements were resolved by discussion with a third reviewer (PD).

### Data Synthesis and Analyses

The data are summarized using a structured narrative description. As there are no standard outcomes for acceptability or feasibility measures for TASs, we include any acceptability and feasibility measures they report. We found that outcomes fell into one of the following categories and thus, these are used as subheadings in the results narrative: comfort, appearance, ease of use, social perceptions, perceptions of alcohol use, barriers/suggestions, the criminal justice system, device tampering, and compliance.

## Results

### Studies in This Review

**Figure 1:**
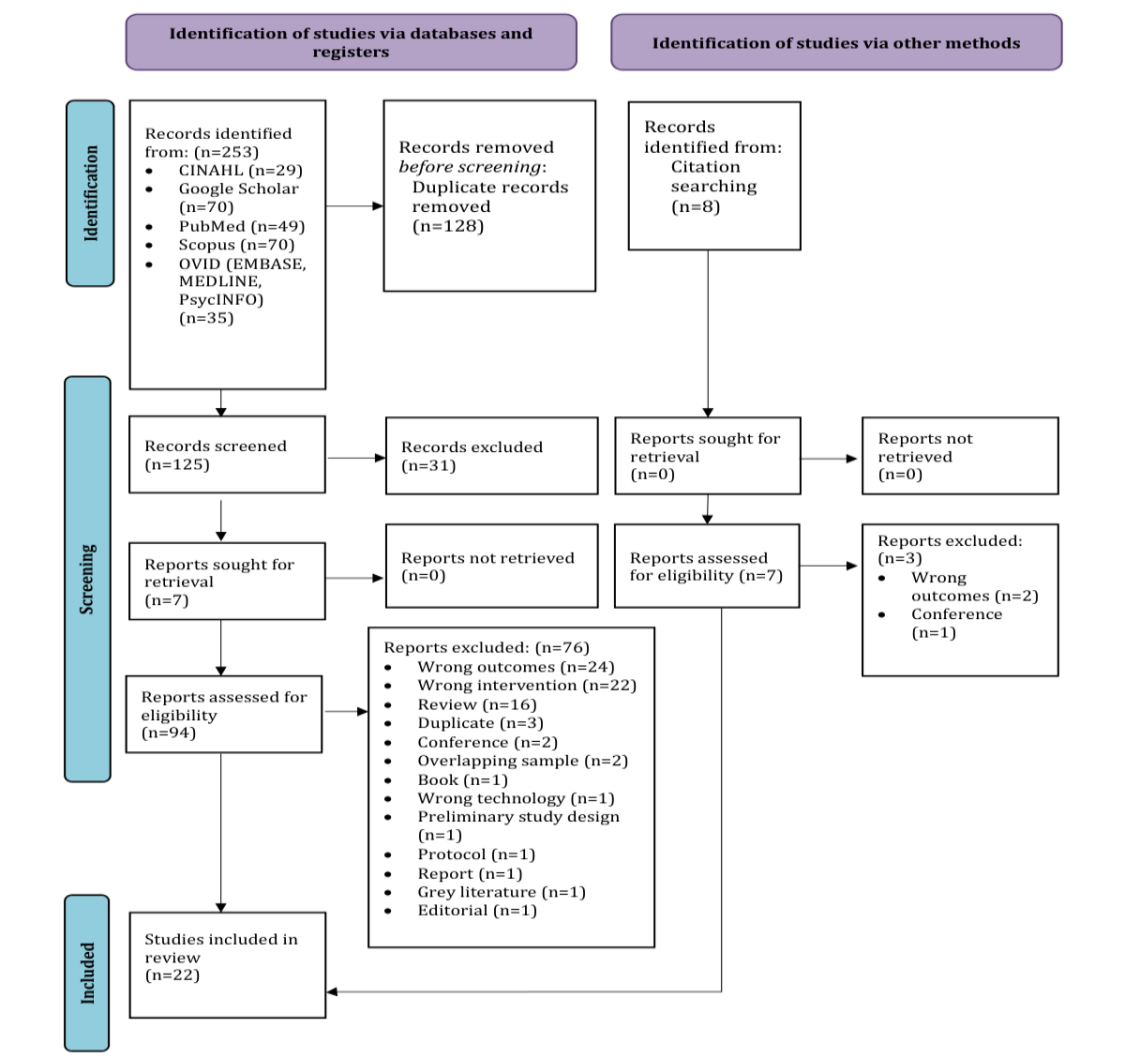
PRISMA-P (Preferred Reporting Items for Systematic review and Meta-Analysis for Protocols) flow diagram.

After removing duplicates, 125 papers were screened, 31 papers were excluded at the title and abstract screening, and 94 full-text papers were assessed for eligibility. A total of 76 papers were then excluded. There were 8 additional papers identified by citation searching; of these, 5 were included. The final sample included 22 publications (Figure 1).

### Characteristics of the Studies

Sixteen studies used a version of SCRAM [8,12,14,23-25,29-38], 4 used a version of WrisTAS [39-42], 3 used BACtrack Skyn [7,38,43], and 1 used Quantac Tally [7]. Some studies used more than 1 version or brand of device. Almost half of the included studies (10/22, 45%) aimed to assess how TASs can be used to measure alcohol consumption. Out of the 22 studies, 8 (36%) aimed to assess acceptability, adherence, and feasibility with TASs, and 5 (23%) used TASs to explore their effectiveness in implementing CM for alcohol reduction treatment and evaluating the efficacy of CM for alcohol use reduction. One investigated nonalcoholic energy drinks and one investigated alcohol-related positive mode enhancement and negative mood reduction.

Most studies (18/22, 82%) included participants who were adults in good health. Only 4 out of 22 (18%) studies included participants who were diagnosed with alcohol dependence. These 4 studies recruited participants from community-based clinics receiving alcohol treatment [8,23,24] or were recruited on admission to a general hospital substance abuse unit [42]. Most were conducted in the United States (17/22, 77%). The earliest paper included is from 1992 but the majority (17/22, 77%) of the studies were published since 2015.

There were 821 participants enrolled in total across the studies, with 793 included in the procedure or analysis. Therefore, 28 participants that were enrolled were not included in the results (28/821, 3.4%) (for reasons such as withdrawing or missing data). One paper [7] was still in the early stages of data collection for one of their studies and so these participant numbers were unknown. Not all studies included detailed information on the participants’ age, gender, and ethnicity. Where information was provided, it could be seen that participants’ ages ranged from 18 to 57 years, the majority included both females and males, and for most, Caucasian participants represented a large majority of the sample (see Table 1).

**Table 1:** Papers included in this review.

Author, year	Design	Aim	Population	Device; Participants: N=821 enrolled (N=793 included)	Age (years); Female (%); Caucasian (%)	Mixed Methods Appraisal Tool score (%)
Alessi et al [24], 2019	Ambulatory	Assess how we can measure alcohol consumption with this technology	Clinical: alcohol outpatient	SCRAMx; 66 (63)	Mean: 40.3; 41.3; 58.7	40
Alessi et al [23], 2017	Ambulatory	Assess acceptability, adherence, and feasibility with this technology	Clinical: alcohol outpatient	SCRAMx; 100 (98)	Mean: 42; 48; 59	80
Averill et al [25], 2018	Pilot RCT^a^, Ambulatory	Effectiveness of TAM^b^ in implementing CM^c^ for alcohol reduction treatment in various population groups, evaluating the efficacy of CM reduction in alcohol use	Nonclinical: at risk drinkers	SCRAM; 37 (37)	Mean: 40.5; 0; 86.5	100
Ayala et al [29], 2009	Laboratory	Assessing nonalcoholic energy drinks with TAM	Nonclinical: good health	SCRAM-II; 15 (15)	Range: 22-35; 60; 80	80
Barnett et al [14], 2017	RCT, Ambulatory	Effectiveness of TAM in implementing CM for alcohol reduction treatment in various population groups, evaluating the efficacy of CM reduction in alcohol use	Nonclinical: heavy drinkers	SCRAM-II, SCRAMx; 30 (30)	Mean: 28.9, range: 21-57; 46; 76.7	80
Barnett et al [12], 2011	Ambulatory	Effectiveness of TAM in implementing CM for alcohol reduction treatment in various population groups, evaluating the efficacy of CM reduction in alcohol use	Nonclinical: heavy drinkers	SCRAM; 20 (13)	Mean: 32; 46; 69.2	80
Caluzzi et al [30], 2019	Ambulatory	Assess acceptability, adherence, and feasibility with this technology	Nonclinical: good health	SCRAM; 34 (30)	Range: 18-29; Sample 1: 58, sample 2: 50; Not known	100
Croff et al [39], 2020	Ambulatory	Assess acceptability, adherence, and feasibility with this technology	Nonclinical: good health	WrisTAS-7; 59 (57)	Mean: 18.82, range: 14-24; 100; 73.3	100
Fairbairn et al [31], 2018	Ambulatory	Alcohol-related positive mood enhancement and negative mood reduction study	Nonclinical: social drinkers	SCRAM; 48 (48)	Mean: 22.6, range: 21-28; 50; 56	100
Goodall et al [32], 2016	Focus groups	Assess acceptability, adherence, and feasibility with this technology	Serving offenders	SCRAM (RAM); 12 (12)	Not known; 0; Not known	100
Luczak et al [40], 2015	Mixed design	Assess how we can measure alcohol consumption with this technology	Nonclinical: good health	WrisTAS-7; 32 (32)	Mean: 23.1; 47; 0	80
Mathias et al [33], 2018	Ambulatory	Effectiveness of TAM in implementing CM for alcohol reduction treatment in various population groups, evaluating the efficacy of CM reduction in alcohol use	Nonclinical: good health	SCRAM; 86 (86)	Mean: 38.5, 37.3, 39.3, 42.6 (cycle 1, 2, 3, 4); Cycle 1: 9, Cycle 2: 20, Cycle 3: 6, Cycle 4: 12; Not known	100
Neville et al [34], 2013	Ambulatory	Assess acceptability, adherence, and feasibility with this technology, assess how we can measure alcohol consumption with this technology	Nonclinical: good health	SCRAMx; 60 (53)	Mean: 21.46, range: 18-37; 0; Not known	80
Norman et al [35], 2020	Ambulatory	Assess acceptability, adherence, and feasibility with this technology, assess how we can measure alcohol consumption with this technology	Nonclinical: good health	SCRAM; 14 (14)	Mean: 21.9, range: 18-29; 50; Not known	80
Rash et al [36], 2019	Ambulatory	Assess how we can measure alcohol consumption with this technology	Nonclinical: heavy drinking	SCRAMx; 22 (19)	Mean: 46.8 (CM), 46 (monitoring); CM: 31, monitoring: 33; 23 (CM); 56 (monitoring)	100
Rosenberg et al [43], 2021	Ambulatory	Assess acceptability, adherence, and feasibility with this technology	Nonclinical: good health	BACtrack Skyn; 5 (5)	Mean: 21.6, range: 21.2-22.3; 60; Not known	80
Sakai et al [8], 2006	Ambulatory and laboratory	Assess how we can measure alcohol consumption with this technology	Alcohol dependent and nonalcohol- dependent	SCRAM; 44 (44)	Mean: 32.8, 38.1, 37.5 (lab: no, low, high dose), mean: 43.5, 39.9 (community: NAD^d^, AD^e^); Laboratory study no/low/high dose: 50; community study NAD: 70, AD: 60; Not known	100
Simons et al [41], 2015	Ambulatory	Assess how we can measure alcohol consumption with this technology	Nonclinical: good health	WrisTAS-7; 60 (60)	Mean: 19.57, range: 18-21; 52; 97	80
Swift et al [42], 1992	Laboratory	Assess how we can measure alcohol consumption with this technology	Nonclinical: good health and alcohol dependent	WrisTAS; 15 (15)	Mean: 27, range: 21-40 (controlled); Range: 31-53 (intoxicated); Controlled: 20; intoxicated: 40; Not known	80
Villalba et al [37], 2020	Focus groups	Assess acceptability, adherence, and feasibility with this technology, effectiveness of TAM in implementing CM for alcohol reduction treatment in various population groups, evaluating the efficacy of CM reduction in alcohol use	HIV-related community	SCRAM; 37 (37)	Not known; 51; Not known	100
Wang et al [7], 2019	Ambulatory and laboratory	Assess how we can measure alcohol consumption with this technology	Nonclinical: good health	Quantac Tally, BACtrack Skyn; Still recruiting	Not known; Not known; Not known	20
Wang et al [38], 2021	Ambulatory and laboratory	Assess how we can measure alcohol consumption with this technology	Nonclinical: good health	BACtrack Skyn, SCRAM-CAM; 25 (15)	Range: 36-38 (study 1); Mean 29.5 (study 2); Study 1: 33.3; study 2: 60; Not known	80

^a^RCT: randomized controlled trial.

^b^TAM: transdermal alcohol monitoring.

^c^CM: contingency management.

^d^NAD: non-alcohol dependent.

^2^AD: alcohol dependent

We found that no study defined acceptability and feasibility in terms of their research. One described how they measured protocol feasibility [40] and one described how they measured acceptability and feasibility of the device [43]. Luczak et al [40] report protocol feasibility as the reliability of each component of the protocol, the validity of TAS data, participant compliance, and reactivity. Rosenberg et al [43] report measuring acceptability using the Acceptability of Intervention Measure scale and feasibility with the amount of alcohol monitor data produced and the correlation between device-reported drinking events and drinking events reported by participants. They also used the Feasibility of Intervention Measure scale.

### Quality Assessment

All studies, except for 2 studies [7,24], met a minimum of 4 out 5 MMAT criteria (>80%) (all scores reported in Table 1 and Multimedia Appendix 5). This is due to Alessi et al [24] not providing details about randomization and participant information. Alessi et al [24] included alcohol treatment outpatients who were randomized to usual care in 2 previous studies; these 2 previous studies are not clearly stated. Wang et al’s study [7] was difficult to score due to incomplete data collection, as their study was still ongoing at the time of publication. With the MMAT, exclusion of low methodological quality studies is discouraged [28]. Due to the nature of many studies, blinding of participants or staff was not possible; in some, there were clear differences between groups [34] or CM incentives were provided [25], where the staff were required to know participant allocation. In many studies, there was only 1 group of participants who all completed the same task and so randomization was not required [7,8,12,23,29-33,35-43]. Not all studies provided clear information on participant information, randomization, incomplete outcome data, and selective reporting; therefore, there was potential bias due to limited information [7,12,14,23,24,29,34,35,38,40-43].

### Acceptability Measures

#### Comfort

SCRAM is the device that is the biggest of the various models and typically worn on the ankle. Participants described rarely noticing SCRAM [8,23,25,30,36]; however, there were a few activities when the device was more noticeable, such as bathing, sport, sleep, and the device vibration/size impacting work and clothing choice [12,14,23,25,30,37]. When rating the SCRAM comfort, on average, it was rated as comfortable by wearers [25,36], and in another study, over half of the participants reported adjusting to any initial discomfort [30]. Other studies reported marks on the skin and itching caused by SCRAM [12,23,25]. Other physical side effects were reported as mild to moderate [14,23,30]. Rash et al [36] found reports of skin marks very uncommon with SCRAM; however, in the study of Caluzzi et al [30] participants described the clamp mechanism of SCRAM as constricting. Methods employed to increase comfort were adjustments made by researchers, including tube socks, plasters, medical tape, and supportive shoe wear. Participants felt that heat and dehydration increased discomfort and those with slimmer legs had greater difficulty keeping the device in a comfortable position. For WrisTAS, device marks were reported infrequently [40], and in 1 study, no marks were reported at all [42]. WrisTAS is smaller than SCRAM and is worn on the wrist. One study allowed participants to remove the device (WrisTAS-7) before engaging in activities that were incompatible with the device and drinking (eg, sports participation) and found minimal reactions to the device [41].

#### Appearance

Most of the views on SCRAM’s appearance were negative [25,30,37]. However, some participants described being less concerned with appearance, as they were motivated to change their alcohol consumption and were looking for help [37]. In Wang et al’s study [38], the design of BACtrack was rated positively, reporting that it was lightweight and comparable to a watch.

#### Ease of Use

Alessi et al [23] found there were 62 adjustments required across 39 participants (N=100 participants). Of these, 56 out of 62 (90%) adjustments were because the device strap was too loose or tight; 3 out of 62 (5%) adjustments were requested by Alcohol Monitoring Systems (AMS) Inc, now better known as SCRAM Systems, and 3 out of 62 (5%) adjustments were requested by both the participants and AMS [23]. Another study reported needing to adjust, reinitialize, or replace the device 5 times in a sample of 19 participants [36]. To download SCRAM data remotely, a home phone landline or cellular signal-based modem is required. Participants who did not have one had to visit family and friends and avoid interruption of personal calls when the modem was connecting to the server to download data, thereby increasing user burden [25]. If this was also not an option for participants, data can be downloaded in-person. One study specifically asked participants to rate the ease of use of WrisTAS; the average rating was 1.2 (scale of 1-10, 1=very easy-to-use) and 26 out of 31 (84%) reported they were confident using the device after the initial session [40]. When asked about the use of Skyn compared to a breathalyzer, the results showed that Skyn was more acceptable, and the most liked features were the ease of use and design [38].

#### Social Perceptions

There were reports that friends and family reacted positively to SCRAM [30,36]. However, a minority reported negative judgements and noted that the ability to hide the device under clothing was appreciated. On hotter days, some chose to wear long trousers to cover the device. This was also reflected with recruitment during the hottest months of summer being difficult [25,37]. Some participants, especially those who had previous links to the criminal justice system, reported feeling embarrassed wearing the device, feared others seeing the device, and were concerned about police harassment [37]. Negative attention from others to the device in 1 study caused 2 participants to withdraw [12]. However, participants in another study rated the social discomfort of SCRAM more moderately at 4.59 (scale of 1-10, 10=extremely uncomfortable) [14]. One study, conducted in a music festival setting with healthy adults, found that most participants did not mind others seeing; some wore long socks, but most did not hide it. Generally, reactions were positive; however, 1 participant asked for the device to be removed due to concerns about not being served in bars [30].

#### Perceptions of Alcohol Use

A frequent comment was that wearing the device worked as a reminder and motivator to not drink alcohol [7,23,25,32,34]. Averill et al [25] using qualitative data suggested that participants believed wearing the device helped reduce drinking and this was supported by quantitative findings. Reduction in alcohol consumption was also rated as the most common personal advantage. Although 1 study found that participants reported purposefully increasing their drinking habits to provide more research data for the device, they also noted this was hard to maintain throughout [30].

#### Barriers, Suggestions, and Future Research

Suggested improvements by researchers and wearers included a smaller size [23,30], being waterproof, improving comfortability [12,14,23,25,38], adjustable straps [30], more notifications about data uploads from the device [38], more information about their transdermal alcohol concentration feedback [12], longer battery life [38], the use of motion or environmental sensors to corroborate output for BACtrack Skyn, and device algorithms to evaluate when deviations in recorded transdermal alcohol concentration are due to environmental factors [38].

#### Criminal Justice System

Goodall et al [32] conducted focus groups with serving offenders in a Scottish prison and found positive views—almost all believed there was an association between alcohol consumption and their offence. Participants reported that knowing someone was monitoring would be an incentive to reduce alcohol consumption, in addition to being constructive in knowing that there would be a consequence if they did consume alcohol. Another suggestion from the focus group was to link wearing the device to a reduced sentence.

### Feasibility Measures

#### Device Tampering

Five studies specifically mention participants tampering with SCRAM [14,23,24,33,36]. Tamper alerts are signaled by AMS if the infrared sensor detects a deviation of 12% above or 17% below the established baseline. Of those 5 studies, 2 report very similar findings [23,24], confirmed tampering in approximately 2% of cases (105 days out of 5017 days of collected data, total participants enrolled: N=66 [24], 139 days out of 6950 days of collected data, total participants enrolled: N=100 [23]), and around half of those tamper days coincided with participants drinking. Confirmed tampers, not linked to drinking, were inadvertently caused by discomfort and subsequently repositioned. Rash et al [36] found 7 events of tampering occurred in 3 participants, most coinciding with drinking. For both studies by Alessi et al [23,24], participants were clinical alcohol treatment outpatients, and in Rash et al [36] the sample consisted of heavy drinkers attending soup kitchens.

Mathias et al [33] were concerned that some of the data suggested instances of tampering. These instances coincided with evidence of alcohol consumption (transdermal alcohol concentration>0.2) and AMS confirmed tamper events (AMS provide independent monitoring of data for the temperature sensor, an infrared sensor, and continuously conducts diagnostic tests to confirm device function). From this, they were able to retrospectively look at data and amend it for future collection. In this study, data influenced CM; therefore, it was important to ensure withholding CM when target behavior did not occur [33]. Barnett et al [14] found that 5.5% of the data were missing due to bracelet removal due to device malfunction and tampering. AMS was able to detect specific tampering instances and the research staff were able to identify and remove specific data.

#### Compliance

A few studies noted 100% compliance [8,29,31,39,43]. Compliance was defined for these studies as no dropouts, no device removal, no missed appointments or assessments, and all participants wore the device for the entire intervention. Other studies mention reasons for not complying, including relocation, events incompatible with continuing (court appearance for driving under alcohol influence, incarceration, day surgery), personal discomfort, concern about employability/negative attention, and device malfunction or inconvenience [12,14,23,24,30,35,36,42]. Although all participants were asked not to remove the devices for any reason other than showering, removal while intoxicated was detected by the temperature sensor [42]. Studies found during recruitment that not everyone was willing to use the devices. Alessi et al [24] found 1 in 10 declined and Alessi et al [23] in another study found that 56 out of 595 (9.4%) participants declined due to SCRAM. Averill et al [25] found that recruitment slowed in summer, and this was suggested to be because the heat would make SCRAM more inconvenient. Compliance was high in the 4 studies with diagnosed alcohol-dependent individuals, one with 100% [8], one with 95% [24], and one with 84% [23] compliance. One does not report any participant dropouts, but device removal was detected [42].

## Discussion

This review aims to assess TAS acceptability and feasibility to objectively monitor alcohol consumption in clinical and nonclinical populations. We identified 22 studies that used or investigated the attitudes and experiences of people using TAS devices. Although the available data do suggest that TAS devices are acceptable, feasible, and have the potential to monitor objective alcohol consumption data, only a few studies were conducted with clinical populations (4 out of 22 studies) or had a specific focus on acceptability and feasibility measures (8 out of 22 studies). We investigated the acceptability outcomes of wearing the TAS, including the comfort, social comfort, appearance, and ease of use of TAS. SCRAM and WrisTAS were reported to provide moderate device comfort, social comfort, and high ease of use. BACtrack was also rated highly for appearance and ease of use. However, there were also reports that SCRAM, the biggest and bulkiest device, caused skin irritation. One study [41] tried to overcome potential skin irritation by allowing participants to remove the device (WrisTAS) before physical activities. Having the freedom to adjust and remove the device for occasions such as bathing or physical activity is not typical, but as the irritation due to TASs during specific activities is one of the known issues affecting each brand of TAS at various levels, this could be a method to reduce this consequence on wearers. However, TASs require precise and secure placement for optimal data collection [44-46]; therefore, removability may only be possible after device training and participants must be trusted to replace the device. Device removability could mean that participants take off the device when consuming alcohol, place it on another sober individual, or not replace it at all. Therefore, removal of devices may not be possible in all situations.

Another uncertainty of these devices is the frequency of device malfunction and how this could impact use. If there were multiple WrisTAS malfunctions, this would not be feasible within clinical treatment or the criminal justice system. If the patient was required to return multiple times, there is an increasing burden on the device wearer and staff. Or, if there were multiple data points lost, the benefit of using this device would diminish and not be able to reliably reinforce criminal sentences or research incentives such as CM. In the included studies in this review, reported malfunctions, noise, and missing data for WrisTAS and BACtrack appeared to be much higher than those for SCRAM [9,39,40,47]. However, there are other advantages of WrisTAS and BACtrack, such as a reduced lag time and physical size and appearance [9,10,38,40]. Some individuals chose not to participate specifically due to the devices. Personal preferences for treatment options are to be expected. There were also reports of dropouts, although dropouts are typical within research. Previous research is aware of the potential discomfort [12,14,23,25,30,37]: TASs must be worn tightly on the wrist for optimal data collection and the wrist is a part of the body that is often on show—this inconvenience could be a reason for withdrawal. Although there are these concerns, many studies found high compliance, including those with alcohol-dependent individuals [7,8,23,24,29-31,35,36,39,42,43].

The only device that has the option of landline use or cellular signal–based modem is SCRAM. This may not be possible for some populations without a stable home or landline; however, for other situations where real-time data collection is not required or when regular research visits for data download is not possible, this would be very beneficial as data download can be done by the participant with no staff member present. BACtrack requires regular data downloading at least every 3 days; otherwise, data are wiped on the device. BACtrack download requires download from the device to an Apple-iPhone operating system device by Bluetooth; for some participants, this may be something they own and can be done remotely and then reviewed online by staff. However, in certain populations, only a few may already own smartphones, specifically for BACtrack, iPhones, or an iPhone operating system device with up-to-date software. One study asked participants to complete self-report web-based data collection surveys, and this was rated as easy-to-use; however, not everyone has access to the internet [40]. The newer generation TASs such as BACtrack avoid this burden by not requiring a landline. However, it needs to be considered if target users would need to be provided a smartphone as well as the TAS device.

Qualitative results suggest that wearing the device appeals as an intervention to reduce alcohol consumption due to the knowledge of someone monitoring acting as a motivator to change their behavior [23,25,32,34]. This could be linked to and incorporate behavior change techniques within the intervention design. However, one aspect that requires further research is if alcohol-dependent patients would feel “under surveillance” by wearing this device and if this could negatively impact the trust and rapport they have with their key worker and service staff.

The potential uses of TASs include alcohol treatment and research contexts. When working with individuals currently receiving alcohol treatment or diagnosed as alcohol-dependent, the amount of alcohol consumed may be a lot greater than that investigated within laboratory studies or with healthy adults not diagnosed as alcohol-dependent. The devices may also be worn for potentially longer periods and used by individuals while heavily intoxicated. These differences could bring to light other considerations that would not be measured within laboratory studies using healthy adults and restricted alcohol consumption, under the eye of a research team. Within short-term laboratory studies with volunteers and payment, there is little reason to not comply. Device tampering is a possibility, and the likelihood of this is increased in populations who use devices within treatment and criminal justice settings. Devices typically contain temperature sensors, which can detect if the device has been removed from the skin. The ability to see this allows for discussion with patients and if appropriate, for tampered data to be removed. Testing to see if the device has been removed, placed on another individual, or any other forms of tampering are issues that could become detectable by these devices as technology advances further.

This review highlights a small number of studies investigating the acceptability and feasibility of TAS devices to objectively monitor alcohol consumption and to compare between devices by using more than one device. Given the growth in the use and appeal of this technology, further research is needed to inform interventions and policy guidance [38,48-50]. There is no standardized method for measuring the acceptability and feasibility of TASs, and there is a need for this to facilitate comparison across different devices.

There is a lack of research on the acceptability and feasibility of TASs to objectively monitor alcohol consumption in any setting, let alone within clinical or criminal justice populations. Although the available data do suggest these devices are acceptable and feasible and have the potential to capture alcohol-monitoring data, there is a need for further research within clinical populations by using robust studies outside a laboratory environment, with long-term monitoring periods. With advancements in technology and the evolution of various TASs coming to market, the focus should potentially be more on the common features of these devices rather than specific brands to better establish the potential of this type of technology. We need to further investigate how clinical populations engage with this technology and any changes in their adherence and use over extended periods of time. This can inform if and how these devices can be implemented in clinical treatment settings with or without other treatment.

## Appendix

Multimedia Appendix 1 Detailed information on the following transdermal alcohol sensor devices: SCRAM, WrisTAS, BACtrack, Milo Sensors, and Quantac.

Multimedia Appendix 2 Database searches, screening, and inclusion of studies for acceptability and feasibility review.

Multimedia Appendix 3 Database search terms.

Multimedia Appendix 4 Data extraction form.

Multimedia Appendix 5 Risk of bias Mixed Methods Appraisal Tool score for acceptability and feasibility review.

## Abbreviations

AMS: Alcohol Monitoring Systems
CM: contingency management
MMAT: Mixed Methods Appraisal Tool
PRISMA-P: Preferred Reporting Items for Systematic review and Meta-Analysis for Protocols
TAS: transdermal alcohol sensor
