# Supplementary material for: Acceptability and Feasibility of Wearable Transdermal Alcohol Sensors: Systematic Review
Source: JMIR Hum Factors. 2022 Dec 23;9(4):e40210. doi: 10.2196/40210 (PMC9823584; doi:10.2196/40210)
Supplement: Multimedia Appendix 1 [file humanfactors_v9i4e40210_app1.docx]

**1. TAS devices: SCRAM, WrisTAS, BACtrack, Milo Sensors and Quantac.**

|  | **SCRAM CAM by SCRAM Systems/AMS** | **WrisTAS by Giner, Inc.** | **Skyn by BACtrack** | **ION wearable by Milo Sensors** | **Tally By Quantac** |
| --- | --- | --- | --- | --- | --- |
| **Date available from** | The first prototype completed in 1993.  Market available from 2003.  Nationally rolled out for use in criminal justice system in England and Wales in 2020. | First wrist worn TAS. Limited information on availability. The first published study on use of a TAS from Giner, Inc. was Swift et al. (1992). | Available for research use globally since 2017. | November 2020, beta release of ION launched. Currently available on sale in the US only. | Quantac operated 2016 – 2017. Quantac ceased business operations in 2017. |
| **Company based** | Colorado, United States.  In 2012 SCRAM Systems spread to England, Canada, Australia, and New Zealand. | Massachusetts, United States. | San Francisco, United States. | Santa Barbara, California, United States.  Milo Sensors, Inc. is a Delaware C-Corp. | New York, United States. |
| **Target audience and purpose** | Criminal justice, use with alcohol-related offenders. | Limited information on WrisTAS target. | Commercial use, currently available at $199/month for research purposes only. CE marked. | Subscriptions are $299/month, with beta tester discounts and research packages. | Commercial use. Limited information on Quantac target. |
| **How they work** | Tests the sweat vapours off the skin using an electrochemical fuel cell at 30-minute intervals. This data is transmitted to the SCRAM Wireless Base Station where it is stored and uploaded to SCRAM’s online monitoring software. | Continuously samples vapour off the skin and stores data until downloaded. It uses a proton exchange membrane (electrochemical) sensor measuring the concentration of alcohol molecules (Marques & McKnight, 2009). | Fuel-cell alcohol sensor (same technology as WrisTAS but a newer generation of this technology). | Non-invasive enzymatic detection technology with a diffusion-limiting membrane and a disposable enzymatic sensor cartridge system. The wearable device can pair with a smartphone (Lansdorp et al., 2019). | Fuel-cell based sensor. |
| **Measure TAC** | Data can be downloaded remotely via landline or cellular signal-based modem in the individual’s home. Data can also be downloaded from the device by connecting to a computer. | This data is then downloaded by connecting to a computer. The device can hold around 21 days of data. WrisTAS can sample at preselected intervals of 10s, 2 mins, 5 min, 10 min or 30 min. | Measures TAC at 20 seconds, 1 minute or 5-minute intervals. The device can be paired to an app on a nearby Apple iOS based device. For data download, the Apple device, the data can be downloaded and viewed on an online server. | Data can be downloaded by syncing the device with a smartphone via Bluetooth. The device can store up to 24 hours of continuous data and the rechargeable Lithium-Polymer battery lasts up to 72 hours. | Measures TAC every 30 seconds. Data is transmitted to an Apple iOS-based device via Bluetooth. |
| **Wear** | Ankle, locked on. | Wrist, removable by user. | Wrist, removable by user. | Wrist, removable by user. | Wrist, removeable by user. |
| **Shower/bathing** | Can be worn in the shower but not submerged under water (in a bath). | Must be removed for showering. | Must be removed for showering. | Not recommended for water immersion, but they are working on an improved water resistance version. | Limited information available. |
| **Image of device** | 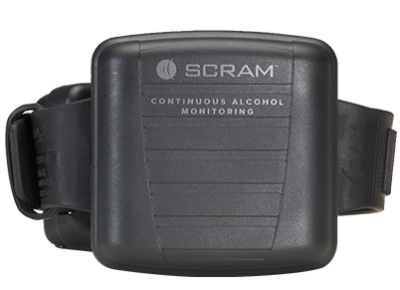 | 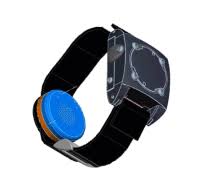 | 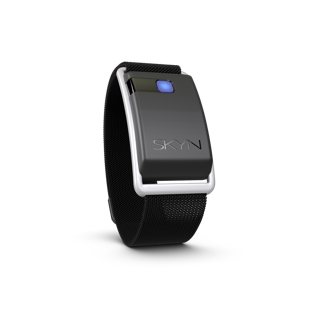<https://skyn.bactrack.com> | 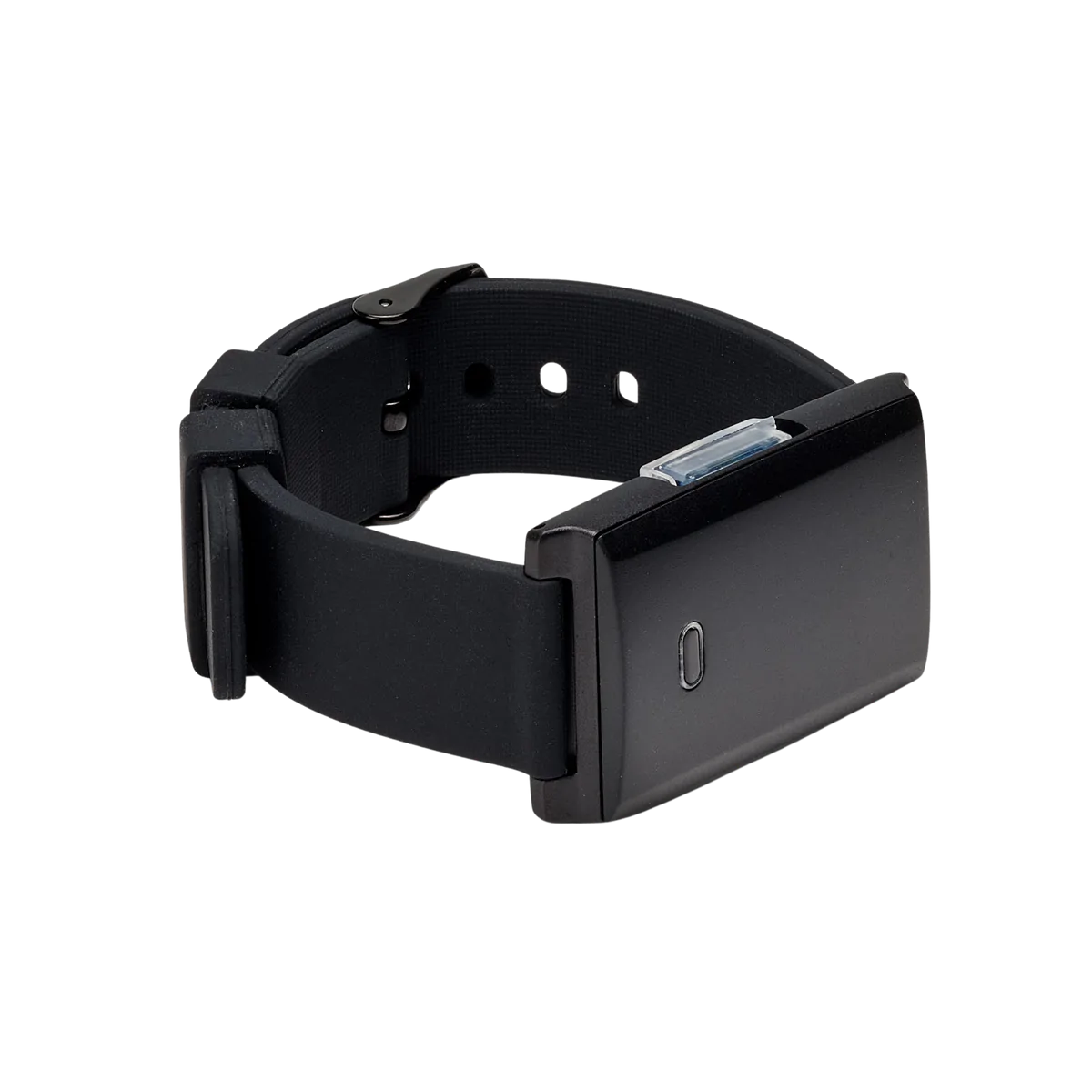 | 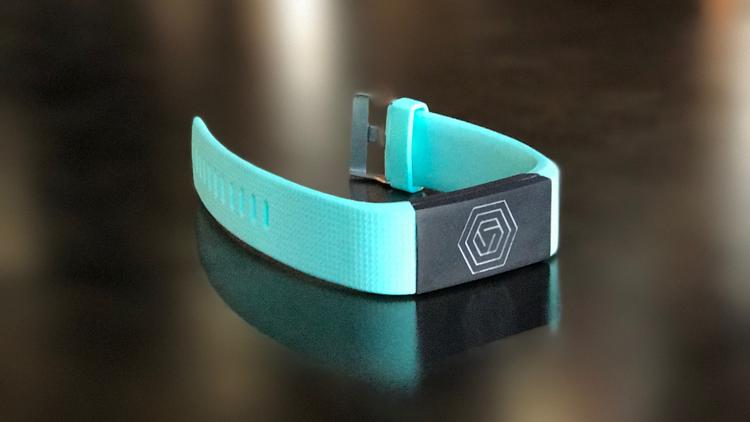 |
