# Supplementary material for: Acceptability and Feasibility of Wearable Transdermal Alcohol Sensors: Systematic Review
Source: JMIR Hum Factors. 2022 Dec 23;9(4):e40210. doi: 10.2196/40210 (PMC9823584; doi:10.2196/40210)
Supplement: Multimedia Appendix 5 [file humanfactors_v9i4e40210_app5.docx]

5. RoB MMAT score for acceptability and feasibility review.

| **Author, Year** | **Total score** | **Criteria 1** | **Criteria 2** | **Criteria 3** | **Criteria 4** | **Criteria 5** |
| --- | --- | --- | --- | --- | --- | --- |
| **Qualitative design** |  | **Is the qualitative approach appropriate to answer the RQ?** | **Are the qualitative data collection methods adequate for the RQ?** | **Are the findings adequately derived from the data?** | **Is the interpretation of results sufficiently substantiated by data?** | **Is there coherence between qualitative data sources, collection, analysis, and interpretation?** |
| Goodall et al. 2016 | 100% | ✓ | ✓ | ✓ | ✓ | ✓ |
| Neville et al. 2013* | 80% | ✓ | ✓ | ✓ | ✓ | ✓ |
| Villalba et al. 2020 | 100% | ✓ | ✓ | ✓ | ✓ | ✓ |
| **Quantitative RCTs design** |  | **Is the randomization appropriate?** | **Are the groups comparable at baseline?** | **Are there complete outcome data?** | **Are outcome assessors blinded to the intervention provided?** | **Did the participants adhere to the assigned intervention?** |
| Alessi et al. 2019 | 40% | ? | ? | ✓ | X | ✓ |
| Alessi et al. 2017 | 80% | ✓ | ✓ | ✓ | X | ✓ |
| Averill et al. 2018 | 100% | ✓ | ✓ | ✓ | ✓ | ✓ |
| Barnett et al. 2017 | 80% | ✓ | ✓ | ✓ | X | ✓ |
| Neville et al. 2013* | 80% | ✓ | ✓ | ✓ | X | ✓ |
| **Quantitative non-RCTs design** |  | **Are the participants representative of the target population?** | **Are measurements appropriate regarding both the outcome and intervention?** | **Are there complete outcome data?** | **Are the confounders accounted for in the design and analysis?** | **During the study period, is the intervention administered as intended?** |
| Ayala et al. 2009 | 80% | ? | ✓ | ✓ | ✓ | ✓ |
| Barnett et al. 2011 | 80% | ✓ | ✓ | X | ✓ | ✓ |
| Caluzzi et al. 2019 | 100% | ✓ | ✓ | ✓ | ✓ | ✓ |
| Croff et al. 2020 | 100% | ✓ | ✓ | ✓ | ✓ | ✓ |
| Fairbairn et al. 2018 | 100% | ✓ | ✓ | ✓ | ✓ | ✓ |
| Luczak et al. 2015 | 80% | ? | ✓ | ✓ | ✓ | ✓ |
| Mathias et al. 2018 | 100% | ✓ | ✓ | ✓ | ✓ | ✓ |
| Norman et al. 2020 | 80% | ✓ | ✓ | ✓ | ? | ✓ |
| Rash et al. 2019 | 100% | ✓ | ✓ | ✓ | ✓ | ✓ |
| Rosenberg et al. 2021 | 80% | ? | ✓ | ✓ | ✓ | ✓ |
| Sakai et al. 2006 | 100% | ✓ | ✓ | ✓ | ✓ | ✓ |
| Simons et al. 2015 | 80% | ? | ✓ | ✓ | ✓ | ✓ |
| Swift et al. 1992 | 80% | ? | ✓ | ✓ | ✓ | ✓ |
| Wang et al. 2019 | 20% | ? | ✓ | X | ? | ? |
| Wang et al. 2021 | 80% | ? | ✓ | ✓ | ✓ | ✓ |
| **Mixed methods design** |  | **Is there adequate rationale for using a mixed methods design?** | **Are the different components of the study effectively integrated to answer the RQ?** | **Are the outputs of the integration of qualitative and quantitative results adequately addressed?** | **Are divergences and inconsistencies between qualitative and quantitative results adequately addressed?** | **Do the different components of the study adhere to the quality criteria of each tradition of the methods involved?** |
| Neville et al. 2013* | 80% | ✓ | ✓ | ✓ | X | ✓ |

✓ = Yes

X = No

? = Can’t tell

*Mixed method designs complete the corresponding criteria: Qualitative design, Mixed method design and the corresponding quantitative design.
